# 5-[2-(4-Acetyl­oxyphen­yl)ethen­yl]benzene-1,3-diyl diacetate

**DOI:** 10.1107/S1600536811044722

**Published:** 2011-10-29

**Authors:** Lin Tang, Dongmei Dai, Yanqing Gong, Jialiang Zhong

**Affiliations:** a Zhejiang Pharmaceutical College, Ningbo 315100, People’s Republic of China; b Key Laboratory for Molecular Design and Nutrition Engineering, Ningbo Institute of Technology, Zhejiang University, Ningbo 315104, People’s Republic of China; c State Key Laboratory of Bio-Organic & Natural Products Chemistry, Shanghai Institute of Organic Chemistry, CAS, 345 Lingling Road, Shanghai 200032, People’s Republic of China; d Shanghai Institute of Pharmaceutical Industry, 1320 Beijing Road (West), Shanghai 200040, People’s Republic of China

## Abstract

The title compound, C_20_H_18_O_6_, was prepared from resveratrol {systematic name: 5-[(*E*)-2-(4-hy­droxy­phen­yl)ethen­yl]ben­z­ene-1,3-diol}, which can be isolated from grapes, through triacetyl­ation with using acetic anhydride in pyridine. The two benzene rings are approximately coplanar, making a dihedral angle of 6.64 (14)°, and the three acet­oxy group are located on the same side of the plane. The skeleton of the compound resembles a table with three legs. In the crystal, mol­ecules are linked *via* C—H⋯O interactions, forming inversion dimers. These dimers are further linked *via* C—H⋯O interactions, forming a three-dimensional structure.

## Related literature

For background to this class of compound, see: González-Barrio *et al.* (2006 ▶). For the preparation of the title compound, see: Sarpierto *et al.* (2007 ▶). For a study of its potential use in radioprotective drug development, see: Koide *et al.* (2011 ▶).
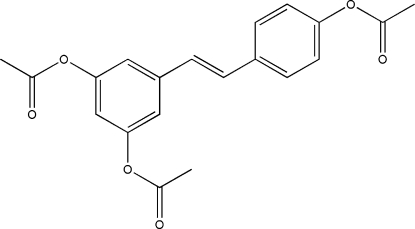


## Experimental

### 

#### Crystal data

C_20_H_18_O_6_
*M* *_r_* = 354.34
Monoclinic, 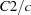

*a* = 31.520 (6) Å
*b* = 6.1211 (12) Å
*c* = 20.110 (4) Å
β = 110.92 (3)°
*V* = 3624.2 (14) Å^3^
*Z* = 8
Cu *K*α radiation
μ = 0.80 mm^−1^
*T* = 296 K
0.23 × 0.10 × 0.08 mm

#### Data collection

Bruker APEXII CCD diffractometer
Absorption correction: multi-scan (*SADABS*; Bruker, 2009 ▶) *T*_min_ = 0.837, *T*_max_ = 0.939
11742 measured reflections
3181 independent reflections
2532 reflections with *I* > 2σ(*I*)
*R*_int_ = 0.020

#### Refinement

*R*[*F* ^2^ > 2σ(*F* ^2^)] = 0.060
*wR*(*F* ^2^) = 0.191
*S* = 1.05
3181 reflections
235 parameters
H-atom parameters constrained
Δρ_max_ = 0.57 e Å^−3^
Δρ_min_ = −0.19 e Å^−3^

### 

Data collection: *APEX2* (Bruker, 2009 ▶); cell refinement: *SAINT* (Bruker, 2009 ▶); data reduction: *SAINT*; program(s) used to solve structure: *SHELXS97* (Sheldrick, 2008 ▶); program(s) used to refine structure: *SHELXL97* (Sheldrick, 2008 ▶); molecular graphics: *SHELXTL* (Sheldrick, 2008 ▶); software used to prepare material for publication: *SHELXTL*.

## Supplementary Material

Crystal structure: contains datablock(s) I, global. DOI: 10.1107/S1600536811044722/rk2305sup1.cif

Structure factors: contains datablock(s) I. DOI: 10.1107/S1600536811044722/rk2305Isup2.hkl

Supplementary material file. DOI: 10.1107/S1600536811044722/rk2305Isup3.cml

Additional supplementary materials:  crystallographic information; 3D view; checkCIF report

## Figures and Tables

**Table 1:** Hydrogen-bond geometry (Å, °)

*D*—H⋯*A*	*D*—H	H⋯*A*	*D*⋯*A*	*D*—H⋯*A*
C20—H20*C*⋯O1^i^	0.96	2.56	3.402 (4)	147
C16—H16*B*⋯O5^ii^	0.96	2.56	3.438 (4)	153
C18—H18*A*⋯O3^iii^	0.96	2.60	3.494 (4)	156
C8—H8*A*⋯O6^iv^	0.93	2.58	3.441 (4)	154

Symmetry codes: (i) 
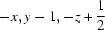
; (ii) 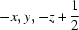; (iii) 
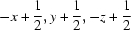
; (iv) 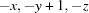.

**Fig. 1.**
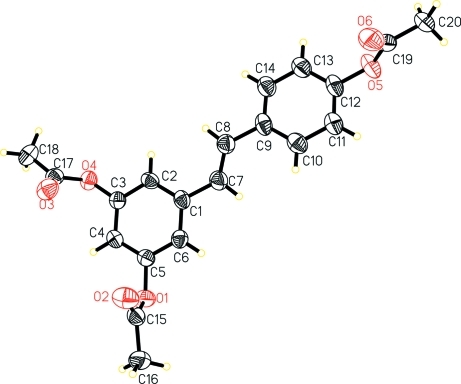
The title molecule structure with the atom numbering scheme. Displacement ellipsoids are drawn at the 30% probability level. H atoms are presented as a small spheres of arbitrary radius.

**Fig. 2.**
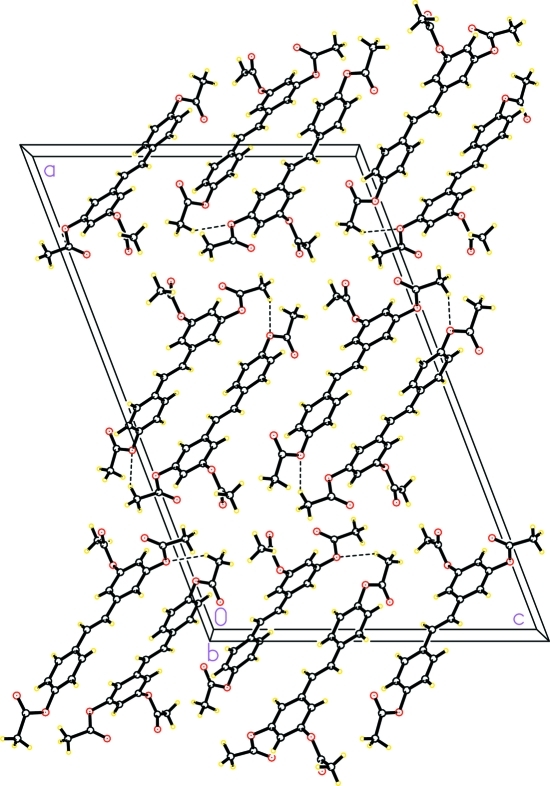
The packing diagram of the title compound, viewed down the b axis.

## supplementary crystallographic information

### Comment

The title molecule, 3,4',5-triacetoxy-*trans*-stilbene (Fig. 1), is the triacetylation product of resveratrol, which can be isolated from grapes (González-Barrio *et al.*, 2006). In the molecular structure of the title compound, two benzene rings were substantially coplanar with dihedral angle 6.64 (14)°. Three acetoxy group located in the same side of the plane. As a result, the whole structure looks like an interesting long table with three legs.

In the crystal, molecules of title compound packed with formation an infinite *Z* form (Fig. 2). Molecules are linked by non-classical C–H···O hydrogen bonds,  which played an important role for the stability of the crystal structure (Table 1).

Koide's study (Koide *et al.*, 2011) showed that the title compound effectively protected the live cells after γ-irradiation and it may be a leading candidate for radioprotective drug development.

### Experimental

The title compound was prepared according to the procedure (Sarpierto *et al.*, 2007) through triacetylation by using acetic anhydride in pyridine (1:1, v/v). Crystals appropriate for X-ray diffraction data collection were obtained from methanol solution, yielding colourless block-like crystals after a week at room temperature.

### Refinement

All H atoms were placed in geometically idealized positions and constrained to ride on their parent atoms with C–H distances of 0.93Å (0.96Å for methyl group) and *U*_iso_(H) = 1.2(1.5 for CH_3_)*U*_eq_(C).

### Crystal data

**Table:**

C_20_H_18_O_6_	*F*(000) = 1488
*M**_r_* = 354.34	*D*_x_ = 1.299 Mg m^−^^3^
Monoclinic, *C*2/*c*	Cu *K*α radiation, λ = 1.54178 Å
Hall symbol: -C 2yc	Cell parameters from 3228 reflections
*a* = 31.520 (6) Å	θ = 4.7–67.0°
*b* = 6.1211 (12) Å	µ = 0.80 mm^−^^1^
*c* = 20.110 (4) Å	*T* = 296 K
β = 110.92 (3)°	Block, colourless
*V* = 3624.2 (14) Å^3^	0.23 × 0.10 × 0.08 mm
*Z* = 8	

### Data collection

**Table:**

Bruker APEXII CCD diffractometer	3181 independent reflections
Radiation source: fine-focus sealed tube	2532 reflections with *I* > 2σ(*I*)
graphite	*R*_int_ = 0.020
φ and ω scans	θ_max_ = 67.0°, θ_min_ = 4.7°
Absorption correction: multi-scan (*SADABS*; Bruker, 2009)	*h* = −37→36
*T*_min_ = 0.837, *T*_max_ = 0.939	*k* = −7→7
11742 measured reflections	*l* = −19→24

### Refinement

**Table:**

Refinement on *F*^2^	Primary atom site location: structure-invariant direct methods
Least-squares matrix: full	Secondary atom site location: difference Fourier map
*R*[*F*^2^ > 2σ(*F*^2^)] = 0.060	Hydrogen site location: inferred from neighbouring sites
*wR*(*F*^2^) = 0.191	H-atom parameters constrained
*S* = 1.05	*w* = 1/[σ^2^(*F*_o_^2^) + (0.1023*P*)^2^ + 2.2789*P*] where *P* = (*F*_o_^2^ + 2*F*_c_^2^)/3
3181 reflections	(Δ/σ)_max_ = 0.001
235 parameters	Δρ_max_ = 0.57 e Å^−^^3^
0 restraints	Δρ_min_ = −0.19 e Å^−^^3^

### Special details

**Table:**

Geometry. All s.u.'s (except the s.u. in the dihedral angle between two l.s. planes) are estimated using the full covariance matrix. The cell s.u.'s are taken into account individually in the estimation of s.u.'s in distances, angles and torsion angles; correlations between s.u.'s in cell parameters are only used when they are defined by crystal symmetry. An approximate (isotropic) treatment of cell s.u.'s is used for estimating s.u.'s involving l.s. planes.
Refinement. Refinement of *F*^2^ against ALL reflections. The weighted *R*-factor *wR* and goodness of fit *S* are based on *F*^2^, conventional *R*-factors *R* are based on *F*, with *F* set to zero for negative *F*^2^. The threshold expression of *F*^2^ > σ(*F*^2^) is used only for calculating *R*-factors(gt) *etc*. and is not relevant to the choice of reflections for refinement. *R*-factors based on *F*^2^ are statistically about twice as large as those based on *F*, and *R*-factors based on ALL data will be even larger.

### Fractional atomic coordinates and isotropic or equivalent isotropic displacement parameters (Å^2^)

**Table:**

	*x*	*y*	*z*	*U*_iso_*/*U*_eq_	
O1	0.16481 (6)	0.7332 (3)	0.46756 (9)	0.0754 (5)	
O2	0.21472 (8)	0.5358 (4)	0.43718 (12)	0.0992 (7)	
O3	0.21702 (7)	1.1732 (4)	0.29763 (12)	0.0906 (6)	
O4	0.14826 (6)	1.2873 (3)	0.29000 (10)	0.0714 (5)	
O5	−0.11728 (7)	0.1955 (4)	−0.02189 (12)	0.1001 (7)	
O6	−0.07902 (11)	0.0003 (4)	−0.07410 (13)	0.1110 (8)	
C1	0.08924 (7)	0.7665 (4)	0.28105 (13)	0.0645 (6)	
C2	0.10294 (7)	0.9669 (4)	0.26198 (12)	0.0646 (6)	
H2A	0.0887	1.0215	0.2162	0.077*	
C3	0.13752 (7)	1.0835 (4)	0.31104 (12)	0.0600 (5)	
C4	0.15881 (7)	1.0105 (4)	0.37985 (12)	0.0620 (6)	
H4A	0.1815	1.0918	0.4130	0.074*	
C5	0.14503 (8)	0.8119 (4)	0.39738 (12)	0.0617 (6)	
C6	0.11048 (8)	0.6919 (4)	0.34968 (13)	0.0642 (6)	
H6A	0.1015	0.5605	0.3638	0.077*	
C7	0.05373 (8)	0.6257 (5)	0.23138 (15)	0.0767 (7)	
H7A	0.0469	0.4975	0.2502	0.092*	
C8	0.03135 (9)	0.6616 (5)	0.16494 (15)	0.0761 (7)	
H8A	0.0389	0.7872	0.1456	0.091*	
C9	−0.00525 (8)	0.5237 (5)	0.11593 (16)	0.0741 (7)	
C10	−0.01673 (9)	0.3156 (5)	0.13435 (16)	0.0803 (8)	
H10A	0.0004	0.2548	0.1781	0.096*	
C11	−0.05336 (9)	0.2001 (5)	0.08785 (16)	0.0792 (7)	
H11A	−0.0613	0.0633	0.1000	0.095*	
C12	−0.07737 (9)	0.2943 (5)	0.02379 (16)	0.0772 (7)	
C13	−0.06570 (10)	0.4942 (5)	0.00422 (17)	0.0835 (8)	
H13A	−0.0823	0.5524	−0.0402	0.100*	
C14	−0.02968 (10)	0.6077 (5)	0.05012 (16)	0.0808 (7)	
H14A	−0.0217	0.7424	0.0366	0.097*	
C15	0.19909 (8)	0.5858 (4)	0.48039 (14)	0.0699 (6)	
C16	0.21382 (11)	0.5035 (6)	0.55443 (17)	0.0948 (9)	
H16A	0.2381	0.4006	0.5623	0.142*	
H16B	0.1887	0.4330	0.5621	0.142*	
H16C	0.2242	0.6235	0.5870	0.142*	
C17	0.18971 (9)	1.3158 (4)	0.28572 (12)	0.0668 (6)	
C18	0.19501 (12)	1.5425 (5)	0.26272 (19)	0.0925 (9)	
H18A	0.2247	1.5590	0.2602	0.139*	
H18B	0.1914	1.6449	0.2965	0.139*	
H18C	0.1724	1.5698	0.2167	0.139*	
C19	−0.11458 (13)	0.0548 (5)	−0.07061 (14)	0.0847 (8)	
C20	−0.16046 (14)	−0.0225 (7)	−0.11694 (17)	0.1184 (14)	
H20A	−0.1575	−0.1227	−0.1518	0.178*	
H20B	−0.1785	0.1002	−0.1406	0.178*	
H20C	−0.1749	−0.0949	−0.0883	0.178*	

### Atomic displacement parameters (Å^2^)

**Table:**

	*U*^11^	*U*^22^	*U*^33^	*U*^12^	*U*^13^	*U*^23^
O1	0.0720 (10)	0.0922 (12)	0.0612 (10)	0.0095 (9)	0.0228 (8)	0.0065 (8)
O2	0.0966 (14)	0.1150 (17)	0.0855 (14)	0.0354 (13)	0.0319 (12)	0.0047 (12)
O3	0.0806 (12)	0.1032 (15)	0.1010 (15)	0.0186 (11)	0.0483 (11)	0.0178 (11)
O4	0.0629 (9)	0.0650 (10)	0.0877 (12)	0.0039 (7)	0.0287 (8)	0.0061 (8)
O5	0.0743 (12)	0.1182 (17)	0.0978 (15)	−0.0211 (11)	0.0186 (11)	−0.0451 (13)
O6	0.141 (2)	0.1095 (18)	0.0868 (15)	−0.0075 (16)	0.0461 (15)	−0.0264 (12)
C1	0.0513 (11)	0.0761 (15)	0.0679 (14)	−0.0055 (10)	0.0235 (10)	−0.0116 (11)
C2	0.0519 (11)	0.0831 (16)	0.0554 (12)	0.0072 (11)	0.0150 (10)	−0.0011 (11)
C3	0.0544 (11)	0.0611 (12)	0.0664 (13)	0.0046 (10)	0.0239 (10)	−0.0005 (10)
C4	0.0534 (11)	0.0670 (14)	0.0632 (13)	−0.0008 (10)	0.0178 (10)	−0.0098 (10)
C5	0.0565 (12)	0.0715 (14)	0.0577 (12)	0.0044 (10)	0.0212 (10)	−0.0025 (10)
C6	0.0607 (13)	0.0670 (14)	0.0685 (14)	−0.0035 (10)	0.0275 (11)	−0.0031 (11)
C7	0.0627 (14)	0.0930 (18)	0.0749 (16)	−0.0060 (13)	0.0252 (12)	−0.0037 (14)
C8	0.0691 (15)	0.0825 (17)	0.0778 (17)	−0.0064 (13)	0.0277 (13)	−0.0042 (13)
C9	0.0584 (13)	0.0822 (17)	0.0881 (18)	−0.0114 (12)	0.0338 (13)	−0.0290 (14)
C10	0.0642 (14)	0.097 (2)	0.0773 (16)	0.0033 (13)	0.0220 (12)	−0.0157 (14)
C11	0.0681 (15)	0.0801 (17)	0.0874 (19)	−0.0074 (12)	0.0255 (14)	−0.0200 (14)
C12	0.0637 (14)	0.0838 (18)	0.0844 (18)	−0.0133 (13)	0.0266 (13)	−0.0318 (14)
C13	0.0751 (16)	0.096 (2)	0.0774 (17)	−0.0051 (14)	0.0248 (14)	−0.0176 (14)
C14	0.0764 (16)	0.0869 (18)	0.0805 (18)	−0.0104 (14)	0.0299 (14)	−0.0170 (14)
C15	0.0592 (13)	0.0694 (14)	0.0734 (15)	−0.0003 (11)	0.0143 (11)	0.0024 (12)
C16	0.0807 (18)	0.110 (2)	0.085 (2)	0.0081 (17)	0.0194 (15)	0.0267 (17)
C17	0.0672 (14)	0.0792 (16)	0.0565 (12)	0.0017 (12)	0.0253 (11)	−0.0013 (11)
C18	0.104 (2)	0.088 (2)	0.102 (2)	−0.0070 (17)	0.0569 (19)	0.0091 (16)
C19	0.114 (2)	0.0813 (18)	0.0563 (14)	−0.0215 (17)	0.0277 (15)	−0.0038 (13)
C20	0.147 (3)	0.118 (3)	0.0649 (17)	−0.054 (2)	0.0076 (19)	−0.0127 (17)

### Geometric parameters (Å, °)

**Table:**

O1—C15	1.360 (3)	C9—C14	1.372 (4)
O1—C5	1.409 (3)	C9—C10	1.409 (4)
O2—C15	1.182 (3)	C10—C11	1.392 (4)
O3—C17	1.188 (3)	C10—H10A	0.9300
O4—C17	1.351 (3)	C11—C12	1.367 (4)
O4—C3	1.397 (3)	C11—H11A	0.9300
O5—C19	1.330 (4)	C12—C13	1.375 (4)
O5—C12	1.403 (3)	C13—C14	1.369 (4)
O6—C19	1.195 (4)	C13—H13A	0.9300
C1—C6	1.379 (3)	C14—H14A	0.9300
C1—C2	1.399 (4)	C15—C16	1.481 (4)
C1—C7	1.481 (4)	C16—H16A	0.9600
C2—C3	1.380 (3)	C16—H16B	0.9600
C2—H2A	0.9300	C16—H16C	0.9600
C3—C4	1.379 (3)	C17—C18	1.491 (4)
C4—C5	1.378 (3)	C18—H18A	0.9600
C4—H4A	0.9300	C18—H18B	0.9600
C5—C6	1.378 (3)	C18—H18C	0.9600
C6—H6A	0.9300	C19—C20	1.490 (5)
C7—C8	1.288 (4)	C20—H20A	0.9600
C7—H7A	0.9300	C20—H20B	0.9600
C8—C9	1.484 (4)	C20—H20C	0.9600
C8—H8A	0.9300		
			
C15—O1—C5	117.03 (19)	C11—C12—C13	122.0 (3)
C17—O4—C3	118.62 (19)	C11—C12—O5	120.0 (3)
C19—O5—C12	118.9 (2)	C13—C12—O5	117.7 (3)
C6—C1—C2	118.5 (2)	C14—C13—C12	120.0 (3)
C6—C1—C7	117.5 (2)	C14—C13—H13A	120.0
C2—C1—C7	123.9 (2)	C12—C13—H13A	120.0
C3—C2—C1	120.0 (2)	C13—C14—C9	120.3 (3)
C3—C2—H2A	120.0	C13—C14—H14A	119.8
C1—C2—H2A	120.0	C9—C14—H14A	119.8
C4—C3—C2	121.8 (2)	O2—C15—O1	123.0 (2)
C4—C3—O4	120.6 (2)	O2—C15—C16	126.0 (3)
C2—C3—O4	117.4 (2)	O1—C15—C16	111.0 (2)
C5—C4—C3	117.2 (2)	C15—C16—H16A	109.5
C5—C4—H4A	121.4	C15—C16—H16B	109.5
C3—C4—H4A	121.4	H16A—C16—H16B	109.5
C4—C5—C6	122.3 (2)	C15—C16—H16C	109.5
C4—C5—O1	119.4 (2)	H16A—C16—H16C	109.5
C6—C5—O1	118.2 (2)	H16B—C16—H16C	109.5
C5—C6—C1	120.0 (2)	O3—C17—O4	122.7 (2)
C5—C6—H6A	120.0	O3—C17—C18	126.3 (3)
C1—C6—H6A	120.0	O4—C17—C18	111.0 (2)
C8—C7—C1	127.1 (3)	C17—C18—H18A	109.5
C8—C7—H7A	116.5	C17—C18—H18B	109.5
C1—C7—H7A	116.5	H18A—C18—H18B	109.5
C7—C8—C9	126.9 (3)	C17—C18—H18C	109.5
C7—C8—H8A	116.6	H18A—C18—H18C	109.5
C9—C8—H8A	116.6	H18B—C18—H18C	109.5
C14—C9—C10	118.9 (2)	O6—C19—O5	122.2 (3)
C14—C9—C8	117.6 (3)	O6—C19—C20	126.5 (3)
C10—C9—C8	123.4 (3)	O5—C19—C20	111.3 (3)
C11—C10—C9	120.8 (3)	C19—C20—H20A	109.5
C11—C10—H10A	119.6	C19—C20—H20B	109.5
C9—C10—H10A	119.6	H20A—C20—H20B	109.5
C12—C11—C10	117.8 (3)	C19—C20—H20C	109.5
C12—C11—H11A	121.1	H20A—C20—H20C	109.5
C10—C11—H11A	121.1	H20B—C20—H20C	109.5
			
C6—C1—C2—C3	1.1 (3)	C7—C8—C9—C10	−8.3 (4)
C7—C1—C2—C3	−177.6 (2)	C14—C9—C10—C11	−2.5 (4)
C1—C2—C3—C4	−1.4 (3)	C8—C9—C10—C11	175.7 (2)
C1—C2—C3—O4	−177.03 (19)	C9—C10—C11—C12	0.6 (4)
C17—O4—C3—C4	68.8 (3)	C10—C11—C12—C13	1.4 (4)
C17—O4—C3—C2	−115.5 (2)	C10—C11—C12—O5	−173.5 (2)
C2—C3—C4—C5	1.8 (3)	C19—O5—C12—C11	−91.5 (3)
O4—C3—C4—C5	177.33 (19)	C19—O5—C12—C13	93.4 (3)
C3—C4—C5—C6	−2.1 (3)	C11—C12—C13—C14	−1.6 (4)
C3—C4—C5—O1	−178.2 (2)	O5—C12—C13—C14	173.4 (3)
C15—O1—C5—C4	−98.1 (3)	C12—C13—C14—C9	−0.4 (4)
C15—O1—C5—C6	85.7 (3)	C10—C9—C14—C13	2.4 (4)
C4—C5—C6—C1	1.9 (3)	C8—C9—C14—C13	−175.9 (2)
O1—C5—C6—C1	178.0 (2)	C5—O1—C15—O2	5.8 (4)
C2—C1—C6—C5	−1.3 (3)	C5—O1—C15—C16	−175.1 (2)
C7—C1—C6—C5	177.4 (2)	C3—O4—C17—O3	1.7 (4)
C6—C1—C7—C8	−177.1 (3)	C3—O4—C17—C18	−179.9 (2)
C2—C1—C7—C8	1.5 (4)	C12—O5—C19—O6	4.4 (5)
C1—C7—C8—C9	−177.9 (2)	C12—O5—C19—C20	−176.5 (3)
C7—C8—C9—C14	170.0 (3)		

### Hydrogen-bond geometry (Å, °)

**Table:**

*D*—H···*A*	*D*—H	H···*A*	*D*···*A*	*D*—H···*A*
C20—H20C···O1^i^	0.96	2.56	3.402 (4)	147.
C16—H16B···O5^ii^	0.96	2.56	3.438 (4)	153.
C18—H18A···O3^iii^	0.96	2.60	3.494 (4)	156.
C8—H8A···O6^iv^	0.93	2.58	3.441 (4)	154.
C16—H16A···O3^v^	0.96	2.70	3.185 (4)	112.

Symmetry codes: (i) −*x*, *y*−1, −*z*+1/2; (ii) −*x*, *y*, −*z*+1/2; (iii) −*x*+1/2, *y*+1/2, −*z*+1/2; (iv) −*x*, −*y*+1, −*z*; (v) −*x*+1/2, −*y*+3/2, −*z*+1.
